# Recent Advances in the Use of *Galleria mellonella* Model to Study Immune Responses against Human Pathogens

**DOI:** 10.3390/jof4040128

**Published:** 2018-11-27

**Authors:** Thais Cristine Pereira, Patrícia Pimentel de Barros, Luciana Ruano de Oliveira Fugisaki, Rodnei Dennis Rossoni, Felipe de Camargo Ribeiro, Raquel Teles de Menezes, Juliana Campos Junqueira, Liliana Scorzoni

**Affiliations:** Department of Biosciences and Oral Diagnosis, Institute of Science and Technology, São Paulo State University (UNESP), São José dos Campos, São Paulo 12245-000, Brazil; thatha.this@hotmail.com (T.C.P.); barrosdnapp@yahoo.com.br (P.P.d.B.); lu_ruano@hotmail.com (L.R.d.O.F.); dennisrossoni@hotmail.com (R.D.R.); felipe_c_ribeiro@hotmail.com (F.d.C.R.); raquelmenezes93@gmail.com (R.T.d.M.); juliana@ict.unesp.br (J.C.J.)

**Keywords:** *Galleria mellonella*, in vivo study, experimental model, hemocytes, immune response, melanization, antimicrobial peptides, clotting, free radical production, primary immunization

## Abstract

The use of invertebrates for in vivo studies in microbiology is well established in the scientific community. Larvae of *Galleria mellonella* are a widely used model for studying pathogenesis, the efficacy of new antimicrobial compounds, and immune responses. The immune system of *G. mellonella* larvae is structurally and functionally similar to the innate immune response of mammals, which makes this model suitable for such studies. In this review, cellular responses (hemocytes activity: phagocytosis, nodulation, and encapsulation) and humoral responses (reactions or soluble molecules released in the hemolymph as antimicrobial peptides, melanization, clotting, free radical production, and primary immunization) are discussed, highlighting the use of *G. mellonella* as a model of immune response to different human pathogenic microorganisms.

## 1. Introduction

Over the past decades, murine models have been the gold standard for studying microbial infections. Recently, these models have been at the center of social and ethical concerns in the global scientific community about rationalizing the use of animals for research. Moreover, the use of such animals is expensive and laborious, and requires maintenance of sufficient numbers of animals to obtain statistically relevant data. In response, alternative host models have been used including *Acanthamoeba castellanii* (amoebae), *Artemia salina* (brine shrimp), *Caenorhabditis elegans* (roundworm), *Danio rerio* (zebra fish), *Drosophila melanogaster* (fruit fly), and *Galleria mellonella* (greater wax moth) [1,2,3,4,5,6]. Each model can help elucidate issues in particular areas of interest [7].

When compared with traditional mammalian models, *G. mellonella* larvae have considerable advantages: they are easily and inexpensively obtained in large numbers; and are simple to use and easy to maintain without special laboratory equipment. Additionally, their size simplifies infection which involves only one hind pro leg and a syringe to apply a defined amount of pathogen. Additional advantages are that ethical approval is not needed for their use, and their short life cycle (7–8 weeks) make them ideal for large-scale studies [8,9,10,11]. Finally, *G. mellonella* larvae can survive from 25 to 37 °C which makes this model suitable to analyze microorganism behavior at mammalian temperature and, moreover, to study temperature-dependent virulence factors [7,12,13]. Recently the *G. mellonella* genome has been sequenced [14], and transcriptome and microRNA data are available [15,16]. Investigations using *G. mellonella* as a model span multiple fields. Recently, its use as a model of infection for bacterial and fungal diseases has been widely described, as has its use for screening the efficacy of new antimicrobials [9].

A wide range of microorganisms have been studied using this model including Gram-positive and Gram-negative bacteria such as *Streptococcus pyogenes* (group A streptococcus) [17,18], *Streptococcus pneumoniae* (pneumococcus) [19], *Enterococcus faecalis* [20,21,22,23], *Enterococcus faecium* [24,25], *Staphylococcus aureus* [26], *Listeria monocytogenes* [27,28], *Pseudomonas aeruginosa* [29,30], *Escherichia coli* [31,32], *Klebsiella pneumonia* [33,34], *Legionella pneumophila* [35], *Francisella tularensis* [36], *Acinetobacter baumannii* [37] and various species of *Burkholderia* [38]. Also, a variety of fungal pathogens have been investigated such as *Aspergillus fumigatus* [39], *Histoplasma capsulatum* [40], *Paracoccidioides brasiliensis* and *P. lutzii* [41], *Fusarium species* [42], and other *Cryptococcus species* [43,44]. Importantly, *G. mellonella* was first described as a model for studying virulence of *Candida albicans* isolates to differentiate between pathogenic and non-pathogenic strains [45,46]. A few reports describe the use of *G. mellonella* with viruses, including Tipula iridescent virus (TIV) [47], Nodamura virus [48], densonucleosis virus and iridovirus [49], invertebrate iridescent virus 6 [50], bovine herpes simplex virus-1 (BHSV-1) [51], and mycovirus [52].

The larval immune system of *G. mellonella* exhibits remarkable structural and functional similarities to the innate immune response of mammals [9,11,53,54]. The insect cuticle is a barrier to infection much as is mammalian skin. Hemolymph, which is analogous to mammalian blood, contains immune cells called hemocytes that can be compared to mammalian neutrophils in terms of their ability to phagocytose and kill pathogens by production of superoxides [55,56].

**Figure 1:**
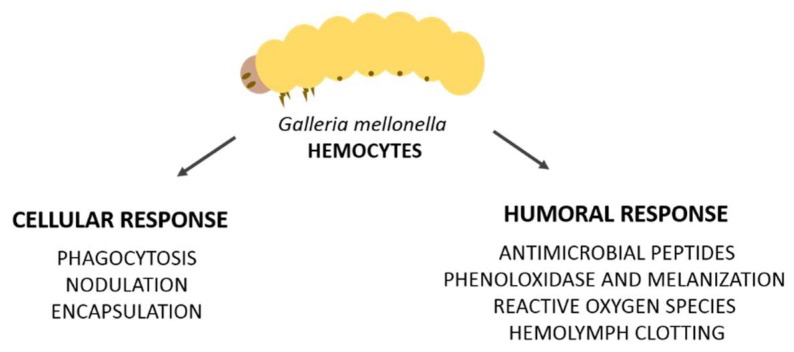
Schematic representation of *G. mellonella* immune response. Hemocyte activity links cellular and humoral responses.

Soluble effector molecules orchestrate humoral responses and include complement-like proteins (opsonins), melanin, and antimicrobial peptides (AMPs) [9]. In this context, the innate immune response of mammals is a vital component in the immune response to pathogenic infections and results obtained using insects show strong correlation with those obtained using mice. *G. mellonella* represents an effective bridge between in vitro studies and studies in mammals [6,57,58]. An important factor to highlight is that humoral and cellular immune defenses are linked (Figure 1); humoral factors influence hemocyte activity and hemocytes are responsible for the production of many humoral molecules [59]. Several mechanistic studies of insect immune response against different pathogens have been reported [60,61], including human pathogens such as *Pseudomonas aeruginosa*, *Enterococcus faecalis*, *Staphylococcus aureus*, *Candida albicans*, *Fusarium oxysporum*, and *Aspergillus fumigatus* [62,63,64,65,66,67].

This review describes the cellular responses of *G. mellonella*, with a focus on the activities of hemocyte: phagocytosis, nodulation, encapsulation, and reactions or soluble molecules released in the hemolymph which are the humoral response (antimicrobial peptides, melanization, clotting, free radical production and primary immunization) to different human pathogens, as described in Figure 1.

## 2. Cellular Immune Response

### Hemocytes and Involved Reactions (Phagocytosis, Nodulation, and Encapsulation)

The cellular immune response of *G. mellonella* is mediated by hemocytes, which circulate freely in hemolymph and also can be found in the digestive tract and fat body of the insect [1,68,69]. During the infection, hemocytes concentration in the hemolymph can vary, with the increase associated with the migration of hemocyte from the organs and tissues to the hemolymph and the decrease relating to the nodulation or encapsulation process [1]. In *G. mellonella* at least six types of hemocytes have been described; the most common types are prohemocytes, plasmatocytes, granulocytes, spherulocytes and oenotocytes [59,70] (Table 1). Plasmatocytes and granulocytes are the most predominant hemocytes in *G. mellonella* and are of great importance in cellular defenses against invasive pathogens and parasites [71].

**Figure 2:**
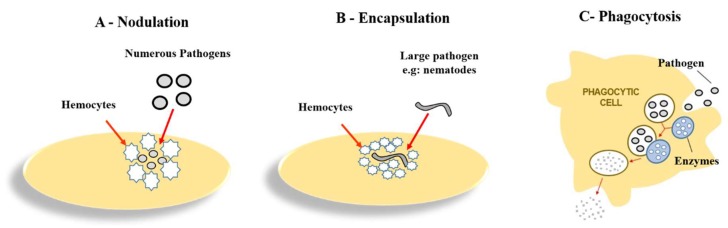
Different defense mechanisms employed by hemocytes in *Galleria mellonella*: (**A**) nodulation: hemocytes form a layer of cells around a group of microorganisms, this process occurs in the presence of a large number of microorganisms [59]; (**B**) encapsulation: plasmocytes and granulocytes form a capsule around large pathogens such as protozoa, nematodes and parasitic insect eggs or larvae [76,77,78]; (**C**) phagocytosis: plasmocytes and granulocytes produce enzymes to destroy the pathogen [1,80].

Hemocytes are responsible for important cellular events: nodulation, encapsulation and phagocytosis [1,54,75]. Hemocytes can act cooperatively in the nodulation process (Figure 2A), forming a layer of cells around multiple microorganisms [59]. This type of defense allows a large number of pathogens to be eliminated from hemolymph. Activation of prophenoloxidase (proPO) and melanization of nodules complete this response [54,59].

Certain pathogens can be killed by encapsulation. In this process, granular cells recognize large foreign bodies such as protozoa, nematodes and parasitic insect eggs or larvae in the hemolymph, promoting plasmacyte binding (Figure 2B). A smooth capsule is formed, composed of superimposed cells [76,77,78]. When capsule formation begins, the number of hemocytes circulating in the hemolymph decreases as they are used in this process. After capsule formation, an increase of hemocytes with enzymatic activity occurs [78]. Encapsulation and nodulation can also occur when phagocytosis fails to eliminate pathogens [79], moreover, the type of response will be determined by pathogen characteristics and concentration.

Another important cellular event is the phagocytosis, in this process enzymes are released by hemocytes through degranulation to destroy the pathogen [1,80]. In *G. mellonella*, phagocytosis (Figure 2C) is mediated by plasmocytes. Granulocytes indirectly contribute to the process [71,81]. Hemocytes phagocytosis is mediated by complement-like proteins which opsonize pathogens and the enzymatic system is activated [54]. Hemocytes also recognize pathogens through receptors called calreticulin or apolipophorin. Calrecticulin is a protein expressed on the surface of hemocytes, and in mammals it is important in cell adhesion, phagocytosis, antigen presentation and in the process of inflammation. The protein apolipophorins III is similar as apolipoprotein E present in mammals and both are associated with innate immunity binding to Gram-positive bacteria and to lipoteichoic acid, as occurs also in mammals promoting phagocytosis [54,82,83,84,85]. Granular cells and oenotocytes produces prophenoloxidase (proPO) and when phagocytic cells encompass a pathogen, this membrane-bound enzyme system is activated (Figure 2C including enzymes such as lectins and lysozymes. This response is similar to the human cellular immune response [1,80]. Lysozymes are antimicrobial peptides (AMPs) that act on the peptidoglycan layer present in fungi and bacteria, and the lectins have the ability to bind to plasmocytes and induce the phagocytosis [1].

When *G. mellonella* is infected with yeast, cellular and humoral immune responses limit microorganism growth [68,86]. A decrease in microorganism occurs due to the nodulation process at the site of infection. Subsequently an increase in the granulocyte density and phagocytosis occurs [68,72,87]. When larvae are injected with phosphate-buffered saline (PBS) or non-virulent pathogens, hemocyte density increases due to the release of bounded prohemocytes [71,72]. These variations in numbers of defense cells change with time of exposure to the pathogen and the virulence of the pathogen [72,88]

Detection and action of the immune system of *G. mellonella* is not specific. Instead of nodulation, the release of antimicrobial peptides (AMPs) and melanization may occur. Actions of cells defense are determined by microbial load, type of pathogen, size and incubation temperature of larvae [40,68]. According to Thomaz et al. (2013) [40], immune responses of *G. mellonella* vary when incubated at different temperatures depending on pathogen. For example, dimorphic fungi have mycelial forms at 25 °C and yeast forms at 37 °C, which results in differentiation of the number of granulomas [40]. Mucormycetes also have virulence dependent to the larvae temperature incubation [89] as well as *Aspergillus terreus* and this virulence increase is associated with growth rate and germination potential [90]. Virulent mechanisms such as capsules, fimbriae and adhesins can damage and kill hemocytes due to cytotoxic or structural effects. [72,91]. Despite the microorganism characteristics, the larvae immune response suffers temperature influence, and larvae incubation at 4 and 37 °C but not at 30 °C have activated the immune system with the increase in hemocyte and AMPs expression [88]. 

Recently, Sheehan and Kavanagh (2018) [68], demonstrated that after 6 h of fungal infection an increase in fungal load and a consequent decrease of hemocytes occurs. After 24 h, a significant increase in proteins associated with tissue degradation caused by fungal proliferation and formation of hyphae is observed. In response to uncontrolled infection, granular cells and AMP are released to achieve control.

In some cases, microorganisms can behave as facultative intracellular pathogens following phagocytosis by hemocytes resulting in intracellular replication and exocytosis. The yeast *Cryptococcus* sp. and the bacteria *Actinobacillus pleuropneumoniae* and *Campylobacter jejuni* serve as examples [86,92,93]. Cellular responses after infection with different microorganisms are summarized in Table 2.

## 3. Humoral Immune Response

### 3.1. Antimicrobial Peptides (AMPS)

*G. mellonella* immune responses consist of two tightly interconnected components, cell-mediated and humoral [54,103]. Components of insect innate immunity include antimicrobial proteins (AMPs) and peptides that are small and cationic molecules which, depending on their mechanism of action, display a broad-spectrum of activity against bacteria, fungi, parasites and viruses [104,105,106]. Mechanisms of action of AMPs range from membrane disintegration to the suppression of intracellular processes, including protein and DNA synthesis [107,108,109,110].

An immune challenge to *G. mellonella* larvae stimulates expression and secretion of a repertoire of structurally and functionally distinct AMPs [15]. Stimulation of invertebrate humoral responses to infecting microbes comprises a range of AMP, which have stimulation and production profiles that vary according to the aggressor agent. Different AMPs are released against a fungal pathogen than AMPs released against a bacterial pathogen [11,65,111].

Insect AMPs are mainly produced in the fat body, hemocytes, digestive tract, salivary glands and reproductive tract [9]. Approximately 20 putative or known defense peptides (moricin-like peptides, cecropins, gloverin, Gm proline-rich peptides 1 and 2, Gm anionic peptide 1 and 2, galiomycin, gallerimycin, inducible serine protease inhibitor 2, 6-tox, defensin, cobatoxin and heliocin-like peptide) are produced by moth larvae [9,109,112,113]. Several have been identified in the hemolymph of immune-challenged larvae [109,114,115,116]. Some immune-relevant proteins and peptides are constantly present in *G. mellonella* hemolymph and their levels can be immune stimulated by a foreign body. These proteins include lysozyme, apolipophorin III (apoLp-III), and anionic peptide 2 [109,116]. Immune-relevant proteins and peptides act in synergy against infection: for example, lysozyme acts synergistically with antimicrobial peptides and proteins [117,118]. These compounds participate in cellular and humoral immune responses and affect host–parasite interactions [51,119,120].

The transcriptome of *G. mellonela* challenge with lipopolysaccharide has identified genes encoding proteins with functions in immunity [15]. This discovery allows the study of alterations in genes encoding antimicrobial peptides that are expressed after infection, and also after drug treatment. Comparing humoral responses to different invading pathogens—Gram-negative and Gram-positive bacteria, *Candida* and filamentous fungi—revealed that kinetics and degree of up-regulation of lysozyme and assorted antifungal peptides vary, depending on the class of the infecting agent. These findings indicate that the immune system of *G. mellonella* is able to distinguish among classes of pathogens [11,116]. The expression profile of AMP-related genes, by quantitative polymerase chain reaction (qPCR) has been widely used to understand the ability of the larval immune system to express certain antimicrobial peptides in response to different types of microorganisms. The most notable characteristics of *Galleria mellonella* AMPs reported in the literature include:

**Gallerimycin**—A defensin-like peptide which is active against filamentous fungi but not against bacteria or yeast [121] even though its expression is strongly induced by bacterial infection. In addition, its expression makes Gram-negative bacteria more susceptible to the action of cecropin-A [106].

**Galiomicin**—Insect defensins, active against 3 filamentous fungi (*Trichoderma viride*, *Fusarium oxysporum* and *Pyricularia grisea*) and 2 yeasts (*Candida albicans* and *Cryptococcus neoformans*) but exhibits no antibacterial activity [122].

**Cecropins**—This peptide is active against Gram-positive and Gram-negative bacteria and against filamentous fungi (*Aspergillus niger*) [115,123]. A synthetic analogue has recently been shown to be active against human pathogenic *Listeria monocytogenes* implicating its potential application in therapeutic strategies [112].

**Cobatoxin**—This cationic peptide is strongly induced in *G. mellonella* following bacterial challenge [121] but it has not yet been characterized in terms of antimicrobial activity. However, cobatoxin may contribute to this potency when paired with AMPs other than Cecropin-A [106].

**Moricins and gloverins**—Moricin-like peptides and gloverins are intriguingly restricted to Lepidoptera. In *G. mellonella*, eight genes are known that encoded 7 different moricin-like peptides. They are particularly active against filamentous fungi and also, to an extent, against yeast, and Gram- positive and Gram-negative bacteria [114]. Gloverins are basic, heat-stable proteins enriched with glycine residues. Five members of the moricin family have been identified [15,109]. They are active against *Escherichia coli*, Gram-positive bacteria, fungi and viruses [10].

Gallerimycin and galiomycin are the most studied AMPs. The gene expression increase of these AMPS was observed with different stimulus as when *G. mellonella* was exposed to mild heat-shock (38 °C) [124], with the pre-exposure to a non-lethal dose of *C. albicans* or its polysaccharide [111], Caspofungin administration [125], prophylactic administration of probiotic bacteria [126] and this increase of expression trigger the insect’s immune response and protect *G. mellonella* larvae against further infection.

### 3.2. Prophenoloxidase and Melanization

The enzyme phenoloxidase (PO), also called tyrosinase, is able to oxidize phenolic substances to quinones that are later converted into the pathway leading to the formation of melanin [127]. Its activity is detected in prokaryotic and eukaryotic organisms and normally this enzyme is found in its inactive form as a proenzyme called prophenoloxidase [10,81,128].

Prophenoloxidase (PPO) is found in *G. mellonella* hemocytes mainly in oenocytoids [129] (these cells originate from an immature prohemocyte and are known to be involved in the immune response [1,130]). It was one of the first immune molecules studied in *G. mellonella*, having been purified in 1995 [127] and characterized in 2012 [131].

During a microbial infection, the PPO cascade is released from oenocytoids and activated by the serine proteases (this cascade is triggered by bacteria or fungi). In its activated form, PO converts tyrosine to dihydroxyphenylalanine (DOPA), and oxidizes phenolic substances to quinones and melanin, leaving infected larvae darkened [132,133,134]. This reaction is also linked to the induced synthesis of antimicrobial peptides and some cellular immune responses [135,136]. According to Gillespie et al. [137], insect PPO cascade activation is similar to the complement system of mammals. As in the innate response of mammals, invading pathogens in the hemolymph of *G. mellonella* are recognized as foreign bodies by proteins and this recognition is followed by the activation of the PPO cascade. These recognition proteins have binding affinity to molecules such as lipopolysaccharides (LPS), peptidoglycan and β-1,3-glucan, which are cell wall components of Gram-negative, Gram-positive bacteria and fungi, respectively [136,138,139,140].

According to Li et al. [141], in unchallenged larvae, components of the phenoloxidase system are kept inactive in oenocytoids and are released after recognition of invasive microbes that initiate the process of melanization. This process occurs during wound healing, and as a part of the cellular immune response. This process is the reason for synthesis and deposition of melanin in infected larvae, which initiates encapsulation of pathogens at the site of injury, followed by coagulation and opsonization and is analogous to the formation of abscesses in mammalian infections [9,10,142].

Several studies have evaluated the melanization process of *G. mellonella* infected by *C. albicans* [68,126,143,144,145]. Borghi et al. [144] evaluated the pathogenicity of 20 clinical strains of *C. albicans* in *G. mellonella* larvae with various fungal doses (from 10^3^ to 10^5^ CFU/larva). They observed some signs of melanization (black spots) starting at the inoculum site after a few hours of fungal infection: however, this process showed a rapid progression related to fungal invasion, leaving dead black colored larvae. Fuchs et al. [145] also showed that *C. albicans*-infected larvae begin to change color to gray within an hour of infection due to melanization and after 24 h of infection most larvae were dead and completely melanized. These data corroborate results from Sheehan and Kavanagh [68] who evaluated early cellular and humoral responses of *G. mellonella* larvae to infection by *C. albicans*. These authors found a significant increase in the expression of the prophenoloxidase-activating proteinase-1 gene (5-fold increase) after 6 h of infection with an inoculum of 5 × 10^5^ cells of *C. albicans*/larvae.

The melanization pattern of *G. mellonella* larvae may also be affected by use of conventional antimicrobials. Some studies used larvae to evaluate different therapeutic strategies [146,147,148]. Alcazar-Fuoli et al. [147] evaluated the effectiveness of voriconazole in *G. mellonella* infected by *Aspergillus fumigatus*. Larvae treated with this antifungal agent showed increased survival, reduced fungal load in hemolymph and in tissue histopathological analysis; the fungus had been almost cleared with tissues appearing healthy, with no visible melanized nodules. Kloezen et al. [148] also correlated levels of melanization with survival of larvae treated with conventional antifungals, amphotericin B and terbinafine.

During *Candida* infections, the phenoloxidase cascade and consequent melanization of the larvae occurs; this response has been associated with β-glucans on the cell wall surface [149]. However, the melanization process is not observed after challenge with *Cryptococcus neoformans*. Trevijano-Contador et al. [86] disputed the correlation of melanization with capsule formation since melanization observed after infection with acapsular mutants of *C. neoformans* was similar to the uninfected group. A possible explanation for this result involves the cryptococcal cell wall that has very low levels of β-glucans compared to *Candida*. Thus, structural differences in the surface of *C. neoformans* determine the type of response elicited by *G. mellonella* [150].

Bacterial infections also cause the melanization of *G. mellonella* larvae. Recently, Jorjão et al. [146] studied the melanization of *G. mellonella* against *Staphylococcus aureus* and *Escherichia coli* infections. These authors verified an increased melanization of larvae after 24 h of the inoculation with *S. aureus* or *E. coli* proving that these bacteria are able to activate the phenoloxidase enzyme. Wand et al. [33] correlated virulence and melanization of *Klebsiella pneumoniae* clinical isolates and reference strains. All strains caused some degree of melanin production compared to the PBS control, but, in general, those strains that caused a greater larval mortality showed higher levels of melanization. The opposite results were also observed. Strains with low larval mortality rates showed little increase in melanin production. Pereira et al. [92] also showed a correlation between pathogenicity of *Actinobacillus pleuropneumoniae* and the degree of larval melanization. In addition, points of melanization around groups of bacterial cells were observed in fat bodies of *G. mellonella* larvae infected with *A. pleuropneumoniae*, thus demonstrating vigorous cellular and humoral immune responses close to the dorsal vessel. With these results, the degree of melanization of the larvae can be a sign of larval health.

### 3.3. Hemolymph Clotting

Hemolymph clotting or coagulation is a complex process essential for hemostasis, healing, and immunity. As in vertebrates, clotting helps in sealing wounds and preventing blood loss [151]. Clotting in *G. mellonella* occurs with the participation of hemocytes, mainly granulocytes [152]. Studies demonstrate that when cells are removed from the hemolymph, the cell-free hemolymph do not clot, this is an evidence of the hemocytes participate in the clotting process [153]. After stimulated, hemocytes are activated and become highly adhesive, functioning in similar fashion to platelets in mammals. These cells are also responsible for other processes of nodulation and melanization, both of which help to defend the host against pathogen invasion [154].

Clotting also involves the participation of soluble factors, including transglutaminase, lipophorin and apolipoproteins, which are all part of a complex cascade [79,153]. The presence of Ca^+2^ is also important for coagulation; chelating Ca^+2^ inhibits coagulation [141]. 

Also, a link between the coagulation cascade and melanization is the activation of the prophenoloxidase cascade [141,155]. These two defense systems are activated by a common protease cascade [155]. Clotting and the prophenoloxidase cascade are activated by the presence of components of microorganism cell walls, such as β-1,3-glucan and lipopolysaccharides, that are important for the immune response of insects [79]. The formation of melanized nodules arrest bacteria; however, it is not known if clotting is also responsible for killing this microorganism [153]. Another factor responsible for the activation of coagulation is the presence of extracellular nucleic acids which are released from damaged tissue and activated oenocytoids [154].

Many advances in the understanding of the clotting cascade in different invertebrates have been made [79,153]; however, its role during infection with human pathogenic microorganisms is still unclear.

### 3.4. Reactive Oxygen Species (ROS)

In invertebrates, hemocytes, cells similar to human phagocytes, exhibiting similar functions, are responsible for the production of reactive oxygen species (ROS) [59].

ROS production in human phagocytes is related to phagocytotic mechanisms and invasive pathogen destruction. Production of these molecules is toxic to pathogenic microorganisms [156,157]. Studies have found that insect species, such as *Drosophila melanogaster* and *G. mellonella*, are able to produce reactive oxygen species [158,159,160]. These molecules have been identified in insect defense systems as cytotoxic agents against pathogens [161,162]. In addition, the presence of homologous components between insect hemocytes and human neutrophils on the ROS generation processes has been reported [55,163,164].

*G. mellonella* ROS production occurs mainly in hemocytes, which are responsible for phagocytosis and pathogen destruction, through the production of superoxide radicals, nitric oxide and hydrogen peroxide [55,159,165]. However, ROS can also be found free in *G. mellonella* hemolymph, because they are produced during melanization through enzymatic oxidation-reduction reactions resulting from the activation of the phenoloxidase cascade, which produces hydrogen peroxide and superoxide [166,167].

Non-selective ROS activity can cause tissue toxicity. In mammals several antioxidant mechanisms degrade ROS to prevent its cytotoxic action on the organism [157,168,169]. Several literature reports have found antioxidant systems in insects that included non-enzymatic and enzymatic components with emphasis on enzymes superoxide dismutase (SOD), glutathione-S-transferase (GT) and peroxidase (P) [170,171,172,173].

Mechanisms of the antioxidant system in *G. mellonella* are also related to the enzymes GT, P and SOD; however, this is not the only mechanism operating in this insect. Some studies have observed antioxidant action of non-enzymatic components in hemolymph, suggesting that antioxidant activity of *G. mellonella* is regulated by two pathways [174,175].

The production of ROS in *G. mellonella* is still unclear; however, it seems to be related to melanization via activation of the phenoloxidase cascade. Enzymatic oxidation-reduction processes on quinones produced in this cascade will generate superoxide anion and, subsequently, other reactive oxygen species. Activation of phenoloxidase can occur in different ways, depending on the type of infecting microorganism, which could result in different responses in the production of ROS. However, no specific studies have been performed to correlate these pathways with the type of pathogenic microorganism [159,176]. Summarizing, specific pathways of ROS production in *G. mellonella* and their correlation with types of infectious microorganisms (bacteria or fungi) are still unclear. Additional research aimed at ROS production and its association with different pathogens is needed.

### 3.5. Primary Immunization 

In invertebrates, no memory and specificity is observed in the immune system which characterizes it as non-specific [177]. One of the functions of adaptive immunity is the ability to develop a memory response. [178]. In invertebrates a phenomenon called “primary immunization” or “primitive immunity” exists which consists of an increase in hemocyte density and expression of antimicrobial peptides (AMPs) that peaks at 24 h after which the response declines [179]. This type of response is verified by exposing the invertebrate to sublethal concentrations of a pathogen. These animals become resistant to subsequent exposure to a higher concentration of pathogen [180]. 

Immune memory can generate a recall response after a lapse in pathogen exposure. This response is a faster and more potent event when compared to the first exposure to microbial stimuli [181]. Some authors accept that the defense of invertebrates against parasites is not completely devoid of induced immune responses, however the mechanisms of this immunizing induction are still unclear [182,183].

Primary immunization is reported in the study of [111] Bergin et al. (2006) that demonstrated that *G. mellonella* larvae can withstand a lethal inoculum of *C. albicans* when previously exposed to a non-lethal dose of this yeast. This response is mediated by an increase in antimicrobial peptide expression. In another study, beta-glucan (polysaccharide present fungal cell wall), was inoculated at different doses into *G. mellonella*, and larvae that received high doses of beta-glucan survived after *C. albicans* infection. High concentrations of glucan induce an increase in hemocyte density and a reduction in yeast proliferation, and they are resistant to subsequent infection [184]. Similar observations were reported by Fallon et al. [185]. Larvae exposed to non-lethal *Aspergillus fumigatus* concentrations increased larvae resistance to a subsequent lethal dose. This resistance was associated with increased hemocyte density and expression of antimicrobial peptides.

## 4. Conclusions

In this review, the cellular and humoral aspects of the immune response of *G. mellonella* larvae when challenged with different human pathogens have been described. Cellular responses and some humoral responses such as antimicrobial peptides and melanization are well defined based on the number of species studied. However, important aspects of the humoral response including clotting, ROS production, and primary immunization still remain unclear. These factors could be important for a better understanding of *G. mellonella* as a model organism to study host–pathogen interactions and the treatment of infections.

## Figures and Tables

**Table 1:** Different types of hemocytes present in hemolymph of larva of *Galleria mellonella* and their main functions.

The Most Common Types of Hemocytes	Morphology and Functions	References
Prohemocytes	Small circular cells with large nucleus. Prohemocytes are not present in all types of insects and are considered progenitor cells.	[54,72,73]
Plasmatocytes	Most common in *G. mellonella*, possessing lysosomal enzymes in the cytoplasm, participate directly in phagocytosis and are also capsule-forming hemocytes.	[54,72,73,74]
Granulocytes	Small nucleus, and granules in the cytoplasm. Participate indirectly in phagocytosis and directly in the encapsulation process. Usually the most common hemocyte type.	[71,73]
Spherulocytes	Present in some insects, it has spherical inclusions. They transport and secrete cuticular components (as the 66 kDa peptide). Its function in insect immunity is not well understood.	[54,72]
Oenotocytes	Large and spherule cells. Are non-adhesive and no phagocytic cells and containphenoloxidase (PO) cascade components.	[54,59,72,73]

**Table 2:** Effect of the human pathogenic microorganisms on hemocyte concentration and phagocytosis. (*) strain-dependent phagocytosis range.

Microorganism	Hemocyte Response after Hours (h) of Infection	Rate of Cells with Phagocytosed Pathogens (%)	Reference
*Candida albicans*	2 h: Decrease 6 h Increase 3 h: Decrease	Not evaluated 4%	[68] [94]
*Candida glabrata*	2 h: Decrease 6 h: Decrease	Not evaluated	[95]
*Candida parapsilosis*	2 h: Increase	5%	[87]
*Candida orthopsilosis*	2 h: Increase	15%	[87]
*Candida metapsilosis*	2 h: Increase	18%	[87]
*Candida krusei*	3 h: Decrease	4%	[94]
*Candida tropicalis*	2 h: Decrease 7 h: Decrease	2 h: 18%	[96]
*Cryptococcus neoformans*	2 h: Increase Not evaluated	20–30% (*) 20–40% (*)	[97] [98]
*Cryptococcus gattii*	Not evaluated	10–40% (*)	[98]
*Aspergillus fumigatus*	2 h: Increase 2 h: Similar to the control 4 h: Increase 24 h: Similar to the control	34% Evaluated but not quantified	[99,100] [63]
*Fusarium oxysporum*	1 h: Decrease	Not evaluated	[101]
*Paracoccidioides brasiliensis*	1 h: Decrease 3h: Decrease	5%	[41]
*Paracoccidioides lutzii*	1 h: Decrease 3 h: Decrease	5%	[41]
*Escherichia coli*	Not evaluated	1 h: 25% 2 h 30%	[102]
